# A case of Lewy body disease and anaplastic astrocytoma presenting with atypical parkinsonism

**DOI:** 10.1111/neup.12848

**Published:** 2022-07-12

**Authors:** Christopher B Leahy, Andrew C Robinson, Edwin Jabbari, Huw R Morris, Imogen Lally, Ibrahim Djoukhadar, Federico Roncaroli, Christopher Kobylecki

**Affiliations:** ^1^ Department of Neurology, Manchester Centre for Clinical Neurosciences, Northern Care Alliance NHS Foundation Trust, Manchester Academic Health Science Centre University of Manchester Manchester UK; ^2^ Division of Neuroscience and Experimental Psychology, Faculty of Biology, Medicine and Health, School of Biological Sciences University of Manchester, Salford Royal Hospital Salford UK; ^3^ Geoffrey Jefferson Brain Research Centre, Manchester Academic Health Science Centre Manchester UK; ^4^ Department of Clinical and Movement Neurosciences UCL Queen Square Institute of Neurology London UK; ^5^ Department of Cellular Pathology Northern Care Alliance NHS Foundation Trust Manchester UK; ^6^ Department of Neuroradiology, Northern Care Alliance NHS Foundation Trust, Manchester Academic Health Sciences Centre University of Manchester Manchester UK

**Keywords:** anaplastic astrocytoma, atypical parkinsonism, Lewy body disease, multiple system atrophy, neuropathology

## Abstract

We report on a patient with atypical parkinsonism due to coexistent Lewy body disease (LBD) and diffuse anaplastic astrocytoma. The patient presented with a mixed cerebellar and parkinsonian syndrome, incomplete levodopa response, and autonomic failure. The clinical diagnosis was multiple system atrophy (MSA). Supportive features of MSA according to the consensus diagnostic criteria included postural instability and early falls, early dysphagia, pyramidal signs, and orofacial dystonia. Multiple exclusion criteria for a diagnosis of idiopathic Parkinson's disease (iPD) were present. Neuropathological examination of the left hemisphere and the whole midbrain and brainstem revealed LBD, neocortical‐type consistent with iPD, hippocampal sclerosis, and widespread neoplastic infiltration by an anaplastic astrocytoma without evidence of a space occupying lesion. There were no pathological features of MSA. The classification of atypical parkinsonism was difficult in this patient. The clinical features and disease course were confounded by the coexistent tumor, leading to atypical presentation and a diagnosis of MSA. We suggest that the initial features were due to Lewy body pathology, while progression and ataxia, pyramidal signs, and falls were accelerated by the occurrence of the astrocytoma. Our case reflects the challenges of an accurate diagnosis of atypical parkinsonism, the potential for confounding co‐pathology and the need for autopsy examination to reach a definitive diagnosis.

## INTRODUCTION

The diagnosis of atypical parkinsonian syndromes and their distinction from idiopathic Parkinson's disease (iPD) is challenging. A definitive diagnosis can often only be established at postmortem examination of the brain.^1^

We report on a patient with a parkinsonian syndrome clinically in keeping with the consensus diagnostic criteria of probable multiple system atrophy (MSA).^2^ Postmortem brain examination identified neocortical predominant Lewy body pathology consistent with iPD and a coexistent diffuse anaplastic astrocytoma (AA) involving the entire cerebral hemisphere, cerebellum, midbrain, and brainstem.

This case report emphasizes the need to look for coexistent conditions in the diagnosis of patients with neurodegenerative diseases where the clinical phenotype is hard to classify.^3^

## CLINICAL SUMMARY

A 70‐year‐old man with a previous history of hypertension and Crohn's disease presented with difficulty walking and impaired upper limb movements. He had dream enactment suggestive of rapid eye movement (REM) sleep behavior disorder and reported difficulties with memory and word finding. He experienced no hallucinations. On examination, he had symmetrical tremor and bradykinesia, which partially responded to levodopa.

Twelve months later, the patient developed severe urinary urge incontinence with associated erectile dysfunction. His mobility declined with backwards falls, and he reported dysphagia with choking episodes. Due to the patient's rapid progression, he was referred to a tertiary movement disorder service for suspected atypical parkinsonism. He was seen for four years following symptom onset and reported ongoing falls. He had persistent symptoms of REM sleep behavior disorder and described sudden onset of sleepiness during the day. He reported difficulty remembering names and with short‐term memory, although he experienced no hallucinations.

On examination at this time, he had facial hypomimia and oromandibular dystonia. Eye examination revealed saccadic hypermetria but normal saccades, with evidence of pretarsal blepharospasm. His speech was dysarthrophonic. There was mild axial rigidity. Both upper limbs were rigid but without significant bradykinesia; there was mild upper limb dysmetria. Limb tendon reflexes were globally brisk, and plantar responses were extensor. His gait was broad‐based and ataxic, with postural instability on the pull test. His blood pressure was 137/79 lying down and 110/72 after 3 min standing.

**Fig 1:**
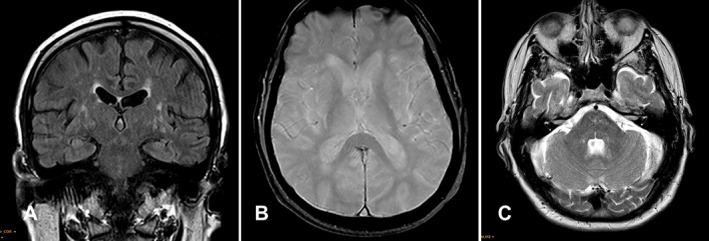
The coronal T2 FLAIR sequence (A) shows widening of perivascular spaces and white matter hyperintensities in the white matter of centrum semiovale and basal ganglia suggestive of cerebral SVD. Axial gradient echo image at the level of the basal ganglia (B) demonstrates a normal signal without signal dropout, and the axial T2‐weighted image (C) at the level of the pons, middle cerebellar peduncles, and cerebellar hemispheres shows patchy non‐specific foci of high T2 signal in the pons.

Brain magnetic resonance imaging (MRI) at this time revealed dilatation of perivascular spaces and white matter T2 hyperintensities in the corona radiata and basal ganglia. There were no specific features of MSA, such as cerebellar atrophy, signal change in the putamen, or the “hot cross bun” sign. In addition, there was no evidence of a space‐occupying lesion or signal abnormality suggesting neoplastic infiltration. The appearances were non‐specific and suggestive mostly of cerebral small vessel disease (SVD) and generalized involution (Fig. 1). Sleep studies did not reveal any evidence of obstructive sleep apnea.

Over the following two years, his mobility worsened further, with deterioration in bowel and bladder control and worsening pretarsal blepharospasm requiring botulinum toxin A injection. Examination revealed marked gaze‐evoked nystagmus and antecollis. He had evidence of moderate limb bradykinesia despite dopaminergic treatment. He was assessed as part of the PROSPECT‐M‐UK study with longitudinal clinical and cognitive assessments. He had no hallucinations. His baseline smell identification score was 1/16. His cognitive assessment six years following onset revealed an Addenbrookes Cognitive Evaluation (ACE)‐III total score of 77/100. One year later, his ACE‐III score was 67/100, with a decline in visuospatial function. The average drop in systolic blood pressure on standing was > 30 mmHg.

The clinical diagnosis was “probable MSA‐parkinsonism type (MSA‐P)” according to consensus diagnostic criteria^2^ with mixed cerebellar and parkinsonian syndrome, incomplete levodopa response, and autonomic failure. Supportive features included postural instability and falls within three years of onset, early dysphagia, pyramidal signs, and orofacial dystonia.^2^

The patient died seven years following the onset of disease. Postmortem examination was limited to the brain.

## PATHOLOGICAL FINDINGS

**Fig 2:**
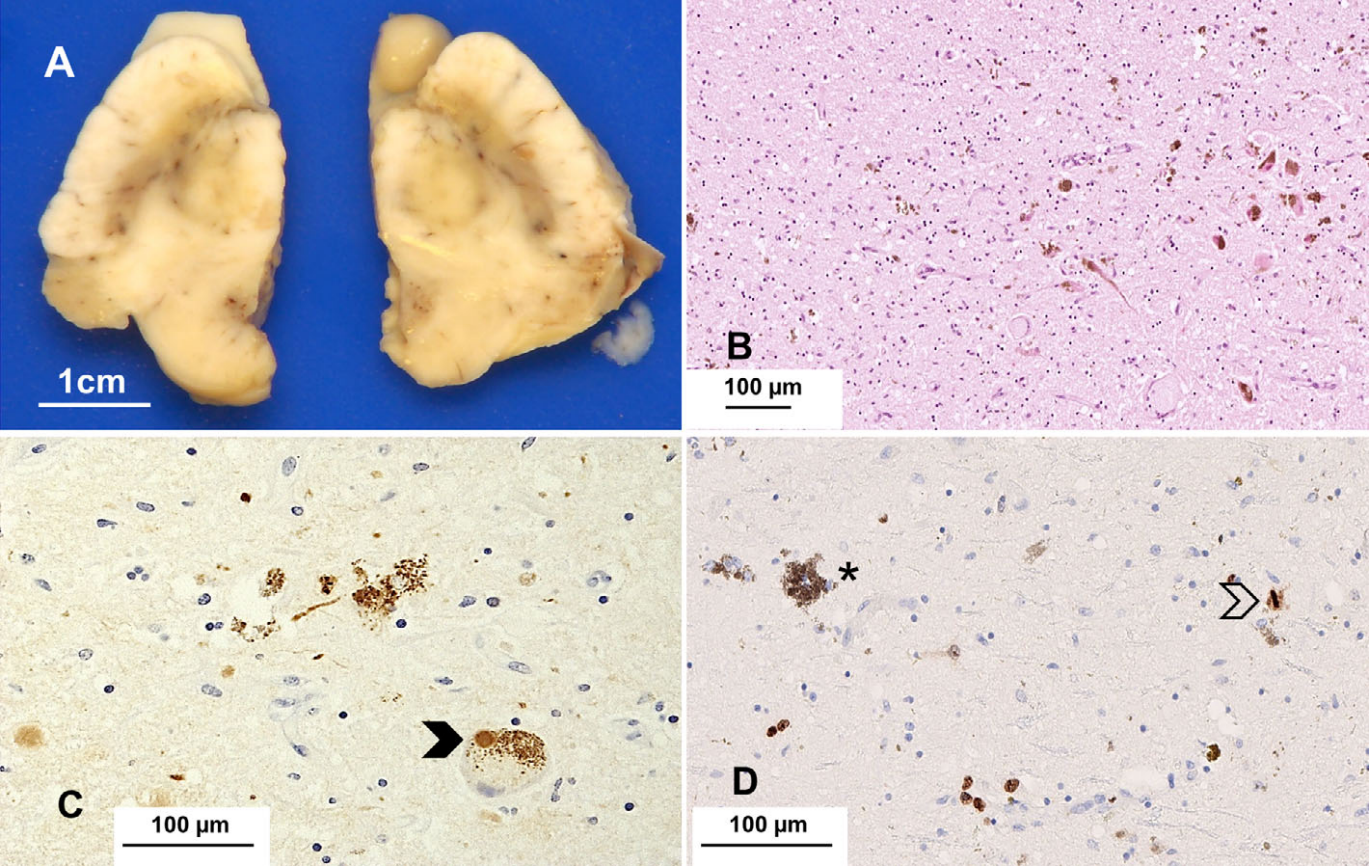
The substantia nigra is pale bilaterally for the age of this patient. Microscopic examination reveals considerable loss of pigmented neurons and pigment incontinence (B, HE – scale bar) and Lewy bodies (arrow) and threads (C, α‐syn); the immunoreaction for Ki‐67 highlights neoplastic cells infiltrating the nigra (asterisk: pigmented neuron); a mitosis is present (arrow) (D, immunoperoxidase). Scale bars: 1 cm (A), 100 μm (B, C, D).

**Fig 3:**
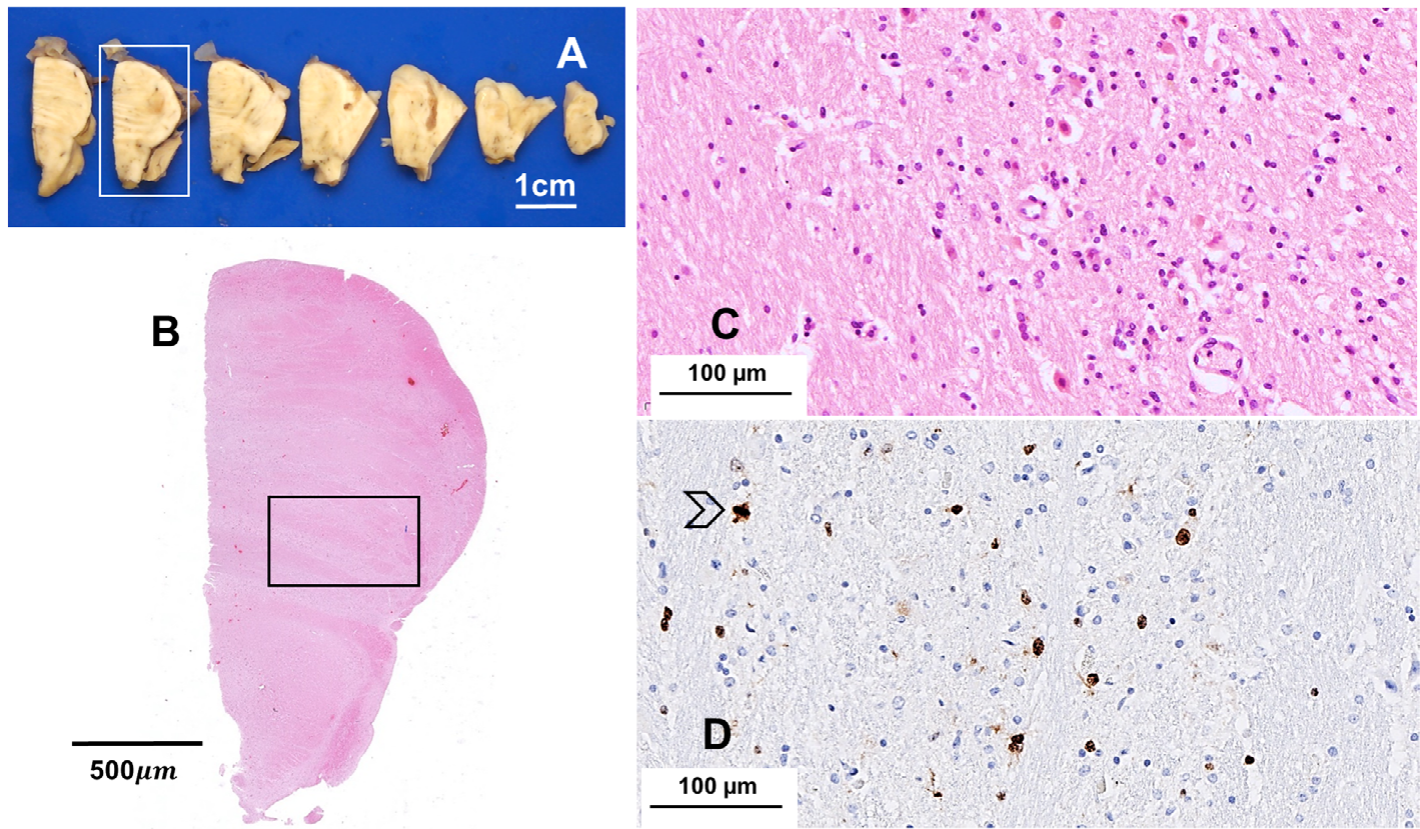
The axial slices of pons and medulla (A) and the whole mount sections of the pons from the framed slice (B, HE) do not show pathological changes of note; the pons shows increased widespread cellularity secondary to infiltration of atypical cells (C, HE; framed areas in the whole mount); tumor cells often express Ki‐67 indicating proliferation; a mitosis is present in this field (arrow) (D, immunoperoxidase). Scale bars: 1 cm (A), 500 μm (B), 100 μm (C, D).

The left cerebral hemisphere and the whole cerebellum and brainstem were dissected following fixation in formalin. Macroscopic examination revealed widening of sulci and loss of volume of both cerebellar hemispheres. Coronal slices did not show obvious pathological features. The substantia nigra was pale bilaterally for the age of this subject (Fig. 2a). The cerebellum, pons, and medulla were macroscopically unremarkable (Figs. 3a).

Microscopic examination revealed neuronal loss and atrophy in all neocortical regions, accompanied by reactive gliosis and mild, focal perivascular inflammation. Leptomeningeal, cortical, and white matter arteries exhibited fibrosis of the walls consistent with moderate SVD. There was bilateral hippocampal sclerosis characterized by neuronal loss, gliosis, and microacceleration in the cornu ammonis (CA)1 sector and subiculum. Neuronal loss in the CA3 and CA2 sectors was much less prominent; the CA4 sector was spared, but there was thinning of the dentate gyrus (Fig. S1). The basal ganglia showed moderate SVD. The substantia nigra showed severe neuronal loss, pigment incontinence, and reactive gliosis (Fig. 2b). Some of the few surviving pigmented neurons contained Lewy bodies. The locus coeruleus was severely atrophic; no Lewy bodies were identified in the hematoxylin–eosin (HE) stained sections.

**Fig 4:**
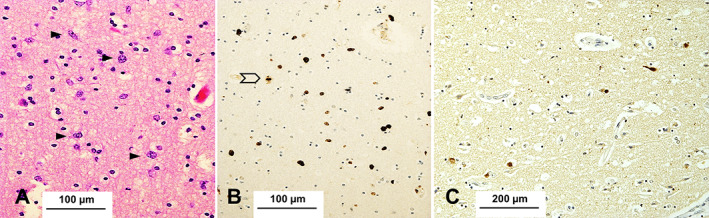
The neocortex (superior temporal gyrus) is infiltrated by atypical cells with irregular, hyperchromatic nucleus and coarse chromatin (arrows) (A); the immunoreaction for Ki‐67 highlights proliferative neoplastic cells; one mitosis is present in this field (arrow) (B). Some neurons contain Lewy bodies (C, α‐syn). Scale bars: 100 μm (A, B), 200 μm (C).

The grey and white matter samples from the cerebral hemisphere (Fig. 4a), cerebellum, midbrain, pons (Fig. 3c), medulla, and to a lesser extent, basal ganglia demonstrated increased cellularity due to diffuse infiltration of atypical cells with scant or fibrillary cytoplasm and irregularly profiled, hyperchromatic nucleus. Mitotic activity was present in all areas examined (Figs. 4a).

The immunoreaction for alpha‐synuclein (α‐syn) (mouse monoclonal, clone 42/α‐syn; BD Bioscience, Franklin Lakes, NJ, USA; 1:500) highlighted Lewy bodies, neuropil threads, and cytoplasmic granular deposits in the nucleus basalis of Meynert, midbrain (Fig. 2c), pons, and medulla. Lewy bodies were present in all neocortical regions (Fig. 4d), while there were fewer in the allocortex, uncus, and amygdala. The cerebellum was spared. Assessment of Lewy body disease (LBD) was performed using Braak and Braak stage and the recently published multi‐institutional consensus criteria.^4^ No Papp–Lantos type oligodendroglial inclusions were present. Tau‐related pathology (mouse monoclonal, clone AT8; Innogenetics, Ghent, Belgium; 1:750) was limited to a few neuropil threads and neurofibrillary tangles in the uncal cortex; there was no amyloid‐beta (Aβ) related pathology (mouse monoclonal, clone 4G8; Cambridge Bioscience, Cambridge, UK; 1:3000). The immunoreaction for TAR DNA binding protein 43 (TDP‐43) (rabbit polyclonal, Proteintech Europe, Manchester, UK; 1:3000) performed on the section of the hippocampus revealed no abnormal inclusions.

The immunoreaction for glial fibrillary acidic protein (GFAP) (mouse monoclonal, clone 6F2; Agilent Dako, Glostrup, Denmark; 1:200) revealed florid reactive astrocytosis in both the grey and white matter. The immunoreaction for ionized calcium‐binding adaptor molecule 1 (Iba1) (mouse monoclonal, clone GT10312; Sigma Aldrich, St. Louis, MI, USA; 1:1000) revealed widespread microglial infiltrates that were more prominent in the white matter. The immunoreaction for isocitrate dehydrogenase 1 (IDH1) R132H mutant protein (mouse monoclonal, clone H09; Dianova, Hamburg, Germany; 1:50) was negative. There was no overexpression of tumor protein p53 (p53) (mouse monoclonal, clone DO‐7; Agilent Dako, Glostrup, Denmark; 1:500). A few T‐lymphocytes were observed around vessels (Cluster of differentiation 3 (CD3), Leica Biosystems, Milton Keynes, UK; 1:250), and only isolated B‐lymphocytes were observed in the white matter and arachnoid (Cluster of differentiation 20 (CD20), Agilent Dako, Glostrup, Denmark; 1:200). The results of representative immunohistochemical stains are revealed in Supplementary Figure S2.

The immunostain for Marker of Proliferation Ki‐67 (Ki‐67) (mouse monoclonal, clone MIB‐1; Agilent Dako, Glostrup; 1:50) revealed several atypical cells (Figs. 2d, 3d, 4c) in keeping with the diagnosis of diffuse astrocytoma.

The source and dilution of antibodies are reported in Supplementary Table S1.

Neuropathological features were in keeping with LBD and neocortical type and consistent with iPD (Braak&Braak stage 6), widespread AA, and bilateral hippocampal sclerosis.

## DISCUSSION

We document a patient with atypical parkinsonism and a clinical phenotype meeting the criteria of MSA‐P. Postmortem examination revealed a Lewy body pathology consistent with neocortical iPD and AA.

The clinical features and disease course were confounded by the coexistent glioma leading to atypical presentation and a clinical diagnosis of probable MSA‐P. It is difficult to comment on the sequence of events in our patient, but we suggest that the AA developed later in the course of iPD. The initial signs and symptoms were due to the α‐synopathy, while rapid progression, ataxia, pyramidal signs, and falls were likely caused and accelerated by neoplastic infiltration of the basal ganglia, midbrain, brainstem and cerebellum. This interpretation is supported by the patient's survival being longer than the average survival of patients with MSA.

According to the 2015 Movement Disorder Society criteria, cerebellar signs in our patient were among the exclusion criteria for a diagnosis of iPD.^5^ Multiple features, including early autonomic failure, recurrent falls, orofacial dystonia, and pyramidal signs, were, conversely, in keeping with MSA. The patient initially responded to levodopa, but his response diminished with time. Early levodopa responsiveness may be seen in MSA and has been documented in more than 50% of patients with autopsy confirmation.^6^ Dementia is an exclusion criterion for MSA; cognitive impairment occurs more frequently in atypical Parkinson's disease (PD) cases diagnosed as MSA in life.^7^

At postmortem, we found extensive infiltration of the cerebral hemisphere, including subcortical grey matter, and midbrain, brainstem, and cerebellum without a space‐occupying lesion. These features would have been in keeping with the diagnosis of gliomatosis cerebri (GC) according to the 2016 World Health Organization (WHO) classification of central nervous system tumours.^8^ GC was initially considered a distinct entity and subsequently regarded as a pattern of diffuse astrocytoma presenting with widespread involvement of the neuraxis.^8^ However, GC is no longer accepted as a variant of diffuse glioma, and the term has been removed from the 5th edition of the WHO classification.^9^ The diagnosis of AA, in this case, was difficult due to the widespread and florid reactive gliosis. Nevertheless, atypical cells with scant or fibrillary cytoplasm, mitotic activity, and noticeable proliferation seen with Ki‐67 that were identified in all regions could not be explained merely by gliosis. Bilateral hippocampal sclerosis was also a feature of this case. The CA1 sector of hippocampus demonstrated moderate neuronal loss associated with reactive gliosis and microacceleration that was disproportionate compared to the density of neurofibrillary tangles, neuropil threads, and amyloid plaques. Hippocampal sclerosis is frequent in the elderly and defines atrophy of hippocampus with more severe damage to the CA1 sector and subiculum. The pathogenesis is unclear. In our patient, hippocampal sclerosis was bilateral, suggesting it could have been related to neurodegeneration rather than SVD.

The association between diffuse astrocytoma (formerly GC) and parkinsonism is exceptional.^1^, ^10^, ^11^, ^12^, ^13^, ^14^ Exclusion features or red flags for a diagnosis of PD were present in all reported patients with diffuse neoplastic infiltration and parkinsonism, including rapid progression, urinary incontinence and frequent pyramidal signs. No patient had a robust sustained levodopa response (Table 1). Diffuse neoplastic infiltration may cause parkinsonism through involvement of the thalamo–cortical pathway or by direct compression of the basal ganglia and nigrostriatal fibres^.1^ Imaging abnormalities may not correlate with the localization on clinical examination.^10^ Equally, tumors involving the basal ganglia may often not be accompanied by parkinsonism. Similar to our case, one patient was diagnosed clinically with MSA due to autonomic failure and cold extremities,^13^ although his presentation at the age of 85 would be atypical for MSA.^2^ We compare the clinicopathological features of the current case and this previously reported case in Table 2. The presence of coexistent Lewy body pathology in our case was the principal difference between the pathological findings, while in neither case were imaging changes diagnostic of GC in life. As mentioned, our patient's survival was longer than that of other reported cases, suggesting that initial symptoms were related to PD pathology, whereas later neoplastic involvement contributed to accelerated disease progression.

**Table 1:** Review of clinical features in pathologically proven cases of diffuse astrocytoma (formerly GC) presenting with parkinsonism (n/a, not available)

Characteristic	Tagliati (2000)^11^	Slee (2006)^12^	Oliviera (2018)^1^	Jang (2013)^10^	Asada (2007)^14^
Clinical diagnosis	Idiopathic Parkinson's disease	Sporadic Creutzfeldt‐Jakob disease	Dementia with Lewy Bodies	Juvenile parkinsonism	Parkinsonism
Levodopa response	Partial	n/a	No	No	No
Pyramidal signs	Present	Absent	Absent	Absent	Present
Cognitive impairment	Present	Present	Present	Present	Absent
Sphincter disturbance	Urinary urgency	Urinary incontinence	Absent	Absent	n/a
Falls < 3y from onset	Yes	No	Yes	No	Yes
MR imaging findings	T2 hyperintensity in left temporal lobe and bilateral thalami	Symmetrical T2 hyperintensity of the thalami, lentiform nuclei, heads of the caudate nuclei.	Diffuse T2 hyperintensity with focal ring‐enhancing nodules	Left parietal T2 high signal	Extensive bilateral diffuse T2 high‐intensity lesions in fronto‐parietal white matter T2 hypointensity in left putamen
Gliomatosis pathology distribution	Pons, midbrain, basal ganglia, amygdala, cortex	Right temporal lobe (biopsy)	Identified on brain biopsy	Identified on brain biopsy	Cerebral white matter (biopsy)

**Table 2:** Comparison between the current case and previously reported case of diffuse glioma mimicking multiple system atrophy (MSA)

Characteristics	Molho et al^13^	Current case
Age of onset (y)	82	70
Levodopa response	Absent	Partial
Pyramidal signs	Present	Present
Cognitive impairment	Present	Present
Sphincter disturbance	Urinary urgency Erectile dysfunction	Urinary incontinence Erectile dysfunction
Falls < 3y from onset	Present	Present
Cerebellar signs	Gait ataxia	Nystagmus Limb and gait ataxia
Duration until death (m)	48	84
MRI findings	Generalized cerebral and pontine atrophy	Dilated perivascular spaces Cerebrovascular disease Cerebellar atrophy
Substantia nigra pathology	Lewy bodies absent Gliomatosis	Lewy bodies present Gliomatosis
Cerebellar pathology	Gliomatosis	Gliomatosis
Basal ganglia pathology	Gliomatosis	Gliomatosis

Neuroimaging features were reviewed for this study. The review confirmed cerebrovascular disease and no definitive imaging features of MSA or widespread glioma, although the subtle signal changes on T2‐weighted sequences in the midbrain and brainstem could still represent early evidence of neoplastic infiltration. Cerebrovascular disease can also mimic MSA,^15^ but the imaging findings were not marked enough in this patient to explain the initial presentation. In the largest series of pathologically confirmed cerebrovascular disease cases mimicking MSA, vascular pathology causing autonomic dysfunction was not seen, and other causes, such as diabetes, were more likely led to autonomic failure.^15^ In our practice, it is usual to perform brain MRI at presentation in cases of suspected atypical parkinsonism, and there were no clinical indications for repeat imaging given the diagnosis and expected progression of MSA. In retrospect, repeat imaging might have been useful to identify more convincing features of neoplastic infiltration.

Our case reflects the challenges of an accurate diagnosis of atypical parkinsonism, and MSA in particular, during life and the contribution of a coexistent condition to the clinical phenotype. In a clinicopathological study of 134 patients, a clinical diagnosis of MSA was only confirmed at postmortem in 83 patients (62%).^1^ Clinical diagnosis of MSA was more likely to be accurate in patients presenting at a younger age. A further study of 203 patients clinically diagnosed as having MSA revealed a diagnostic accuracy of 78%; earlier falls and ataxia were more common in MSA compared to atypical LBD.^7^ Autonomic failure in PD, including postural hypotension meeting criteria for MSA, is increasingly recognized and is associated with more rapid progression and reduced survival.^16^ In retrospect, the abnormal olfactory test supported a diagnosis of PD, as olfaction is typically preserved in MSA.^17^ The visuo‐spatial dysfunction and cognitive decline also supported a cortical LBD. Olfactory testing could be more widely applied to clinical practice to help with differential diagnosis.

The differential diagnosis of patients with poor responsive to levodopa includes progressive supranuclear palsy, corticobasal syndrome, MSA, and vascular parkinsonism. This case emphasizes the importance of considering coexistent conditions, including secondary acquired causes of parkinsonism, such as tumors, which may be difficult to diagnose during life, and the role of postmortem brain examination to confirm the diagnosis in atypical parkinsonism cases.

## DISCLOSURE

CBL, ACR, ID, FR, and IL report no disclosures. EJ reports funding from the PSP Association and the Medical Research Council. CK reports grant funding from the Michael J Fox Foundation and Parkinson's UK. HRM reports research grants from the PSP Association, CBD Solutions, Medical Research Council, and Michael J Fox Foundation.

The funding sources had no role in the design, practice or analysis of this study.

## DISCLOSURE OF ETHICAL STATEMENTS

## APPROVAL OF THE RESEARCH PROTOCOL

The study was approved by Manchester Brain Bank Management Committee (REC reference 19/NE/0242). Under conditions agreed with the Research Ethics Committee, the Manchester Brain Bank can supply tissue or data to researchers without requirement for researchers to apply individually to the REC for approval.

## INFORMED CONSENT

Informed consent was obtained from the patient described in this case report.

## REGISTRY AND THE REGISTRATION NO. OF THE STUDY/TRIAL

n/a

## ANIMAL STUDIES

n/a

## RESEARCH INVOLVING RECOMBINANT DNA

n/a

## PATIENT CONSENT STATEMENT

Informed consent was obtained from the patient described in this case report.

## Supporting information


**Supplementary Table S1** Source, type, antigen retrieval method and dilution of primary antibodies used in immunohistochemistry. (HIER, heat‐induced epitope retrieval).

**Supplementary Figure S1** The whole mount shows HE staining of the left hippocampus. The CA1 sector and subiculum reveal neuronal loss. There is thinning of the dentate gyrus. Scale bar: 500 μm

**Supplementary Figure S2** The temporal neocortex demonstrates reactive astrocytosis (A, GFAP immunoperoxidase) and considerable activation of microglia in the white matter (B, Iba1 immunoperoxidase). The immunoreaction for the mutant protein IDH1 (R132H) demonstrates no expression in tumor cells (C, immunoperoxidase). Only scattered T lymphocytes are seen, mainly in a perivascular location (D, CD3 immunoperoxidase). Scale bars: 50 μm (A), 100 μm (B), 50 μm (C, D).
