# Supplementary material for: A case of Lewy body disease and anaplastic astrocytoma presenting with atypical parkinsonism
Source: Neuropathology. 2022 Jul 12;42(6):540–7. doi: 10.1111/neup.12848 (PMC10084019; doi:10.1111/neup.12848)
Supplement: Supplementary file 1 — Supplementary Table S1 Source, type, antigen retrieval method and dilution of primary antibodies used in immunohistochemistry. (HIER, heat‐induced epitope retrieval). [file NEUP-42-540-s002.docx]

| **Protein** | **Clone** | **Supplier** | **Antibody type** | **Antigen retrieval** | **Primary antibody dilution** |
| --- | --- | --- | --- | --- | --- |
| GFAP | 6F2 | Agilent Dako | Mouse monoclonal | CC1 (76 min, 98°C) | 1:200 |
| Iba1 | GT10312 | Sigma-Aldrich | Mouse monoclonal | CC1 (64 min, 97°C) | 1:1000 |
| IDH1 | H09 | Dianova | Mouse monoclonal | CC1 (52 min, 97°C) | 1:50 |
| Ki-67 | MIB-1 | Agilent Dako | Mouse monoclonal | CC1 (64 min, 95°C) | 1:50 |
| p53 | DO-7 | Agilent Dako | Mouse monoclonal | CC1 (64 min, 95°C) | 1:500 |
| Amyloid-β | 4G8 | Cambridge Bioscience | Mouse monoclonal | 70% Formic Acid (20 min) | 1:3000 |
| Phosphorylated tau | AT8 | Innogenetics | Mouse monoclonal | HIER (20 min, 100°C) | 1:750 |
| Phosphorylated α-synuclein | 42/alpha synuclein | BD Bioscience | Mouse monoclonal | 70% Formic Acid (20 min) | 1:500 |
| TDP-43 | n/a | Proteintech Europe | Rabbit polyclonal | HIER (20 min, 100°C) | 1:3000 |
| CD3 | LN10 | Leica Biosystems | Mouse monoclonal | CC1 (64 min, 97°C) | 1:250 |
| CD20 | L26 | Agilent Dako | Mouse monoclonal | CC1 (64 min, 95°C) | 1:200 |

**Supplementary Table 1:** Source, type, antigen retrieval method and dilution of primary antibodies used in immunohistochemical staining. (HIER – Heat-induced epitope retrieval).
