# Supplementary figures and images for: A case of Lewy body disease and anaplastic astrocytoma presenting with atypical parkinsonism
Source: Neuropathology. 2022 Jul 12;42(6):540–7. doi: 10.1111/neup.12848 (PMC10084019; doi:10.1111/neup.12848)

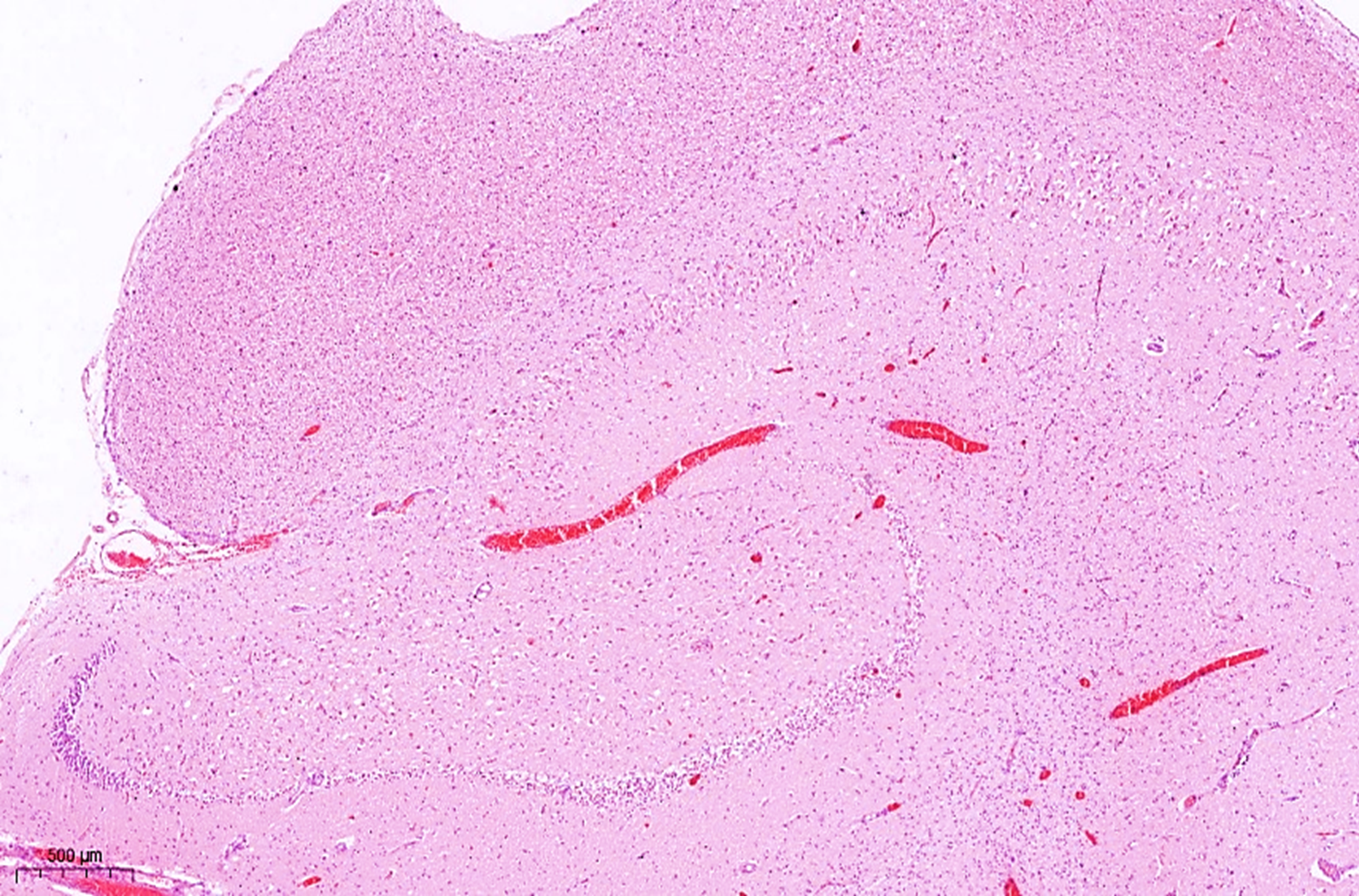

Supplement: Supplementary file 2 — Supplementary Figure S1 The whole mount shows HE staining of the left hippocampus. The CA1 sector and subiculum reveal neuronal loss. There is thinning of the dentate gyrus. Scale bar: 500 μm [file NEUP-42-540-s001.tif]

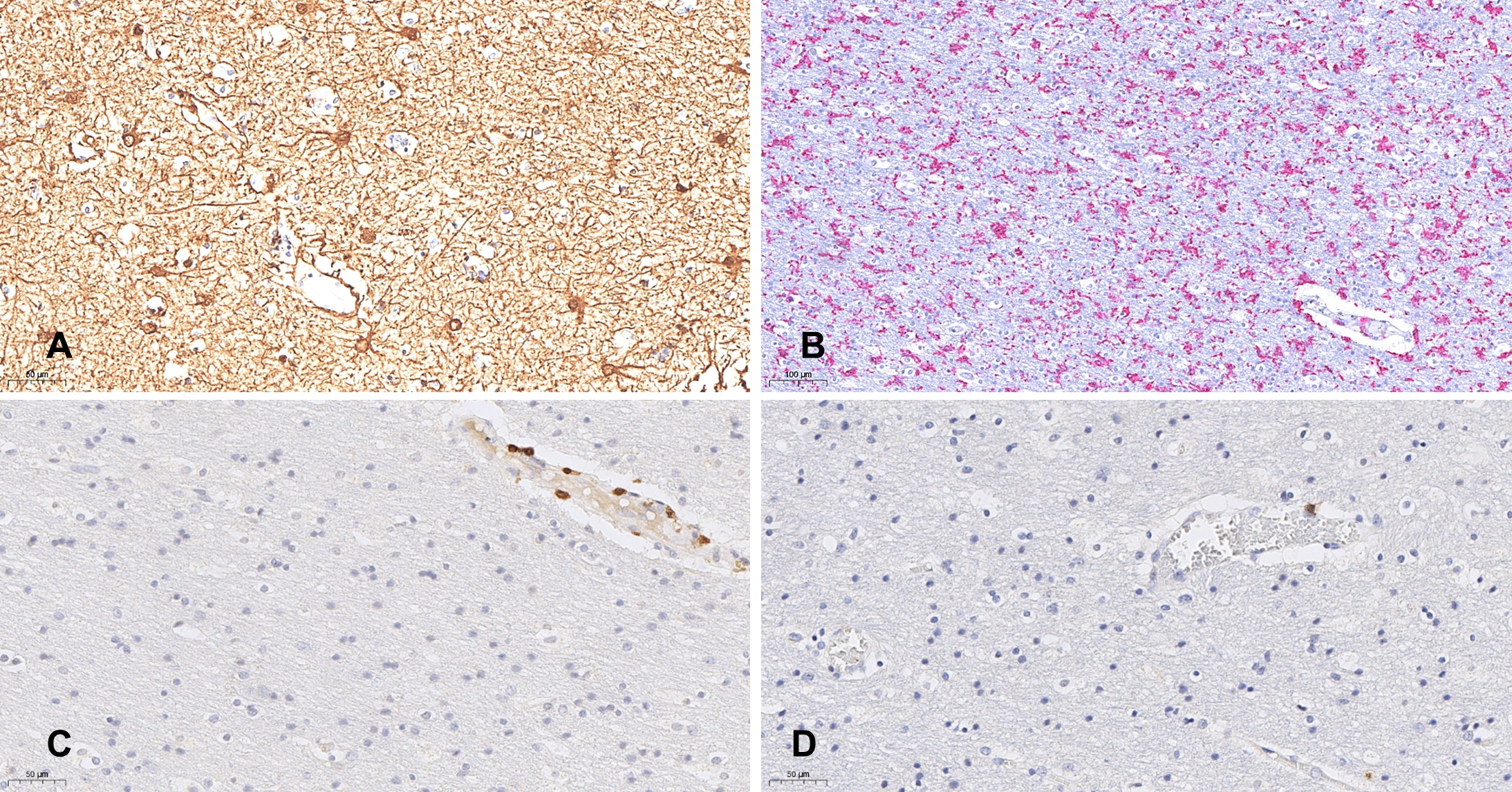

Supplement: Supplementary file 3 — Supplementary Figure S2 The temporal neocortex demonstrates reactive astrocytosis (A, GFAP immunoperoxidase) and considerable activation of microglia in the white matter (B, Iba1 immunoperoxidase). The immunoreaction for the mutant protein IDH1 (R132H) demonstrates no expression in tumor cells (C, immunoperoxidase). Only scattered T lymphocytes are seen, mainly in a perivascular location (D, CD3 immunoperoxidase). Scale bars: 50 μm (A), 100 μm (B), 50 μm (C, D). [file NEUP-42-540-s003.tif]
